# Advances in indoleamine 2,3-dioxygenase 1 medicinal chemistry

**DOI:** 10.1039/c7md00109f

**Published:** 2017-05-16

**Authors:** Alice Coletti, Francesco Antonio Greco, Daniela Dolciami, Emidio Camaioni, Roccaldo Sardella, Maria Teresa Pallotta, Claudia Volpi, Ciriana Orabona, Ursula Grohmann, Antonio Macchiarulo

**Affiliations:** a Department of Pharmaceutical Sciences , University of Perugia , via del Liceo 1 , 06123 Perugia , Italy . Email: antonio.macchiarulo@unipg.it ; Fax: +39 075 585 5161 ; Tel: +39 075 585 5160; b Department of Experimental Medicine , University of Perugia , P.le Gambuli , 06132 Perugia , Italy

## Abstract

Indoleamine 2,3-dioxygenase 1 (IDO1) mediates multiple immunoregulatory processes including the induction of regulatory T cell differentiation and activation, suppression of T cell immune responses and inhibition of dendritic cell function, which impair immune recognition of cancer cells and promote tumor growth. On this basis, this enzyme is widely recognized as a valuable drug target for the development of immunotherapeutic small molecules in oncology. Although medicinal chemistry has made a substantial contribution to the discovery of numerous chemical classes of potent IDO1 inhibitors in the past 20 years, only very few compounds have progressed in clinical trials. In this review, we provide an overview of the current understanding of structure–function relationships of the enzyme, and discuss structure–activity relationships of selected classes of inhibitors that have shaped the hitherto few successes of IDO1 medicinal chemistry. An outlook opinion is also given on trends in the design of next generation inhibitors of the enzyme.

## Abstract 2

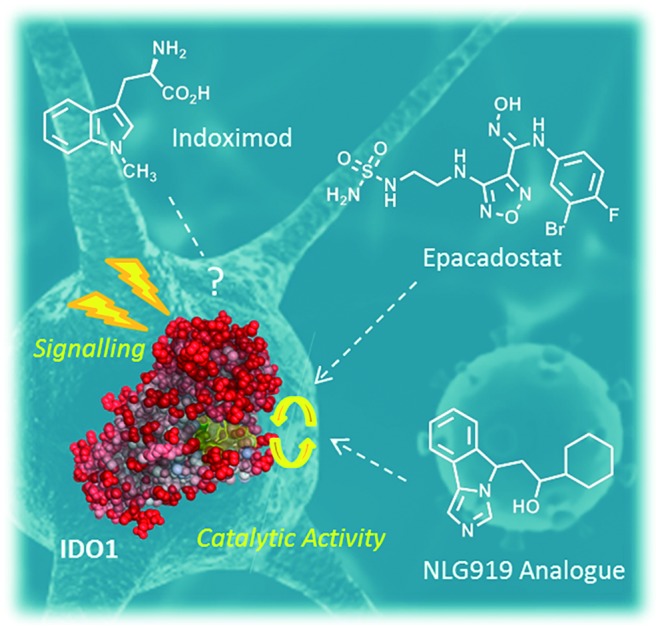
Structure–function relationships of IDO1 and structure–activity relationships of inhibitors are discussed with an outlook on next generation IDO1 ligand.

## Introduction

**Fig. 1:**
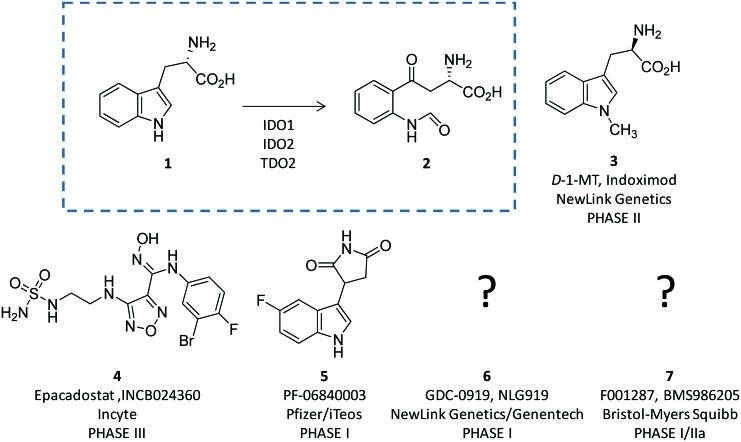
The chemical structures of the substrate (**1**) and product (**2**) of the rate limiting step of the kynurenine pathway are shown in the dashed box. The chemical structures of drug candidates targeting IDO1 (**3–6**) are shown outside of the box. The structures of compounds **5** and **6** are undisclosed.

Indoleamine 2,3-dioxygenases (IDOs) are heme-containing proteins that catalyze the oxidative cleavage of the indole ring of tryptophan (l-Trp, **1**) to produce *N*-formyl kynurenine (**2**) in the first rate limiting step of the kynurenine pathway (Fig. 1).^1^,^2^ The family includes two related enzymatic isoforms, namely IDO1 and IDO2, sharing ∼60% of sequence similarity and featuring distinct biochemical features.^3^,^4^ A third enzyme of the family is the tryptophan-2,3-dioxygenase (TDO2) which is structurally unrelated to IDO1 and IDO2 and is endowed with a more stringent substrate specificity for l-Trp.^5^ Although TDO2 is expressed almost exclusively in hepatocytes where it regulates l-Trp catabolism in response to the diet, IDO1 and IDO2 are widely expressed in macrophages and dendritic cells exerting immunoregulatory functions.^6^ These are accomplished through two major mechanisms including depletion of tryptophan and production of bioactive metabolites along the kynurenine pathway. Specifically, the first mechanism suggests that increasing levels of interferon-γ (IFN-γ) induce IDO1 expression in macrophages and dendritic cells during pathogen infection, leading to consumption of l-Trp and growth arrest of pathogens, whose diet is sensitive to this essential nutrient.^7^ The second mechanism is grounded on production of kynurenine metabolites that bind to the aryl hydrocarbon receptor (AhR), activating signaling pathways that enhance immune tolerance.^8^–^10^ Among the three proteins, IDO1 is the most characterized enzyme and in recent years a second signal-transducing function was revealed for this protein.^11^,^12^ In particular, this signalling function relies on the presence of two immunoreceptor tyrosine-based inhibitory motifs (ITIMs) in the non-catalytic domain of IDO1.^13^ The immunosuppressive cytokine transforming growth factor-β (TGF-β) stimulates phosphorylation of ITIMs by Sarcoma-family (Src-family) kinases and consequent interaction of the phosphorylated enzyme with Src Homology 2 domain Phosphatase-1 (SHP-1) and Src Homology 2 domain Phosphatase-2 (SHP-2), eventually leading to long-term expression of IDO1 and immune tolerance. Conversely, under pro-inflammatory environmental conditions, increasing levels of interleukin-6 (IL-6) trigger the interaction of phosphorylated IDO1 with suppressor of cytokine signalling 3 (SOCS3) that tags the enzyme for proteasome degradation, shortening IDO1’s half-life and promoting inflammatory response.^14^

The breakthrough discovery that IDO1 plays a crucial role in the maintenance of maternal immune tolerance ushered in a great deal of interest in the enzyme, by then considered a master regulatory hub of immunosuppressive pathways in pregnancy, autoimmune diseases, chronic inflammation, and cancer.^15^ In this framework, elevated levels of IDO1 expression found in several tumour cells were associated with the participation of the enzyme in the tumor immuno-editing process which sets up immune tolerance to tumor antigens.^16^,^17^ On this basis, academic groups and pharmaceutical companies have been engaged in the development of IDO1 inhibitors.^18^ Although part of these efforts has proved successful, with a large array of potent and selective inhibitors being disclosed in the literature and patent applications, only few compounds have hitherto entered clinical trials (**3–7**, Fig. 1).^2^,^19^–^22^ In this regard, some studies have highlighted challenges in the development of enzyme inhibitors mostly due to redox properties of the enzyme that may account for the unspecific mechanism of inhibition of many compounds discovered in preclinical studies.^23^,^24^

Starting with an overview on the architecture of IDO1 and its structure–function relationships, in this article we discuss selected classes of inhibitors that have shaped advances in the medicinal chemistry of IDO1, providing outlooks on future trends in the design of next generation compounds.

## Structure–function relationships of IDO1

Since the pioneering work of Sugimoto and coworkers who disclosed the first crystal structure of IDO1 (pdb codes: 2D0T, ; 2D0U),^25^ several other studies have reported additional structures of the enzyme in complex with inhibitors (pdb codes: ; 4PK5, ; 4PK6, ; 5ETW, ; 5EK2, ; 5EK3, ; 5EK4),^26^,^27^ or bearing mutant forms of the protein (pdb codes: ; 4U72, ; 4U74).^28^

**Fig. 2:**
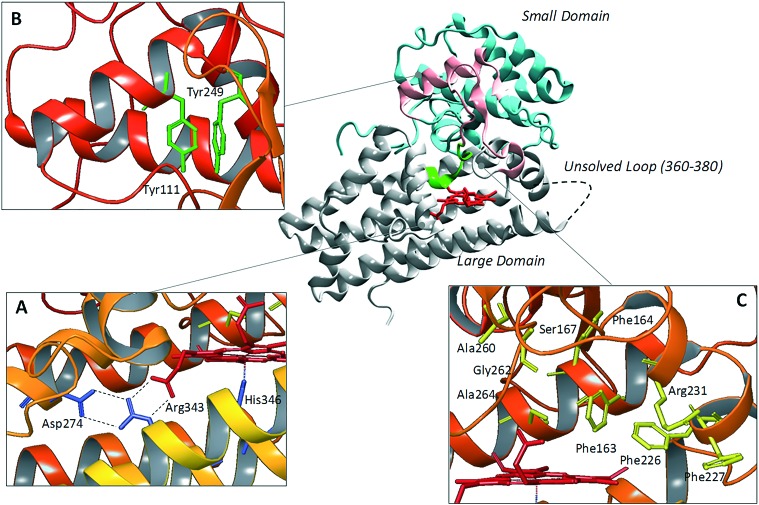
Structure of IDO1 and structure–function relationships depicting residues involved in holding the heme group (box A), ITIM mediated signaling functions (box B), and catalytic activity (box C).

Overall, these structures show a common architecture being composed of a large catalytic domain holding the heme group, and a small non-catalytic domain that is not present in the TDO2 structure (Fig. 2). Specifically, the large domain consists of thirteen α-helices and two 3_10_-helices. Herein, residue His346 provides a coordinative bond to the fifth position of the iron-heme. In agreement with this pivotal role, early mutagenesis experiments of His346 showed a complete loss of the catalytic activity owing to the lack of heme content in IDO1.^29^ In the same study, residue Asp274 was also suggested to be involved in heme binding on the basis of the detrimental effect of Asp274Ala mutation on the catalytic activity. Inspection of the crystal structure reveals an indirect engagement of Asp274 in holding the heme moiety which is achieved through the formation of a salt bridge with Arg343. As a result, the side chain conformation of Arg343 is stabilized by forming hydrogen bonds with one propionate group of the heme cofactor (Fig. 2A).

The small domain is composed of six α-helices, three 3_10_-helices and two β-sheets. It contains two ITIM motifs (Tyr111 and Tyr249) that, upon phosphorylation by Src kinases, promote protein–protein interactions of IDO1 with SHPs or SOCS3, regulating the signalling function of the enzyme (Fig. 2B). Recently, single ITIM-mutated forms of the murine IDO1 protein revealed the distinct and non-overlapping role of the two ITIM motifs, so that ITIM-1 (Tyr115) would associate with SHP phosphatases and ITIM-2 (Tyr253) with the SOCS3 protein.^13^ The large catalytic domain and the small signaling domain are connected to each other by a flexible loop (residues 260–265, Fig. 2C) that shapes the structure of the catalytic pocket above the sixth coordination site of the iron-heme. This highly conserved loop has been thought to be involved in regulating the specificity of substrate recognition and the shift between the catalytically active and inactive conformations of the enzyme which hold the ferrous and ferric forms of the heme group, respectively.^30^,^31^

Although two crystal structures of active site loop mutants Ala260Gly and Gly262Ala (pdb codes: 4UT2, ; 4UT4) were recently disclosed, no publication has yet been reported discussing the impact of such mutations on substrate recognition and IDO1 catalytic activity. However, previous mutagenesis studies have identified residues Phe226, Phe227 and Arg231 as important for substrate binding and catalytic activity of IDO1 (Fig. 2C).^25^ Docking studies and molecular dynamic simulations have further suggested that Phe226 and Arg231 are directly involved in both substrate and inhibitor binding to IDO1, with the former residue providing π–π interactions and the latter residue hydrogen bonds and π–cation interactions with ligands.^32^,^33^ Conversely, Phe227 may indirectly affect substrate binding by engaging Arg231 in a π–cation interaction that suits the enzyme for the catalytic activity. Ser167, Phe163 and Phe164 are further residues of the catalytic pocket that have been studied by means of mutagenesis experiments (Fig. 2C).^34^ Specifically, spectroscopic and kinetic data for mutant Ser167Ala indicated that this residue is not involved in substrate recognition.^35^ However, in a more recent study, mutant Ser167Ala was shown to affect the activity of different enzyme inhibitors, suggesting that it is indeed involved in hydrogen bond interactions with ligands.^36^ A large flexible loop (residues 360–380, the crystal structures of which are not solved) borders the entrance channel to the catalytic site of IDO1. Early coarse graining and molecular dynamic simulations have suggested a role for such a loop in controlling the shuttling of the substrate and products to the catalytic site of the enzyme.^33^,^37^ This hypothesis has been recently supported by mutagenesis experiments and spectroscopic analysis, highlighting a role for Thr379 in forming a hydrogen bond interaction with l-Trp (**1**) that stabilizes a closed substrate-bound conformation of the enzyme.^37^,^38^

**Fig. 3:**
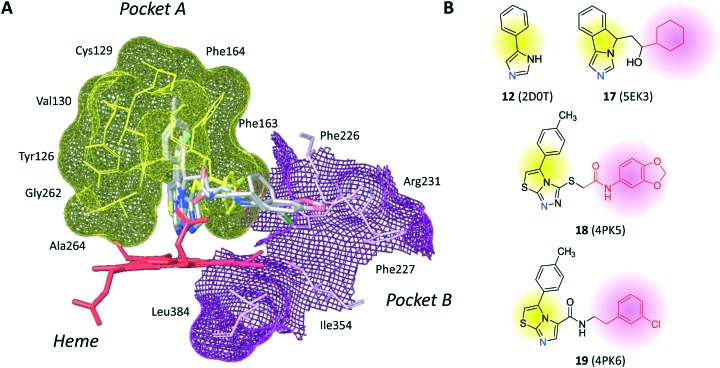
(A) The catalytic cleft of IDO1 is shaped with two pockets: pocket A (yellow surface) is mostly composed of aromatic and hydrophobic residues, while pocket B (magenta surface) is made of one positively charged residue and aromatic residues. (B) Co-crystallized inhibitors (**14**, **19–21**) with shadow colors indicating the part of the chemical structure occupying pocket A (yellow) and/or pocket B (magenta).

The inspection of inhibitor-bound complexes of IDO1 has unveiled additional features of the enzyme that affect the molecular recognition of small molecules.^26^,^27^ Specifically, two pockets in the catalytic cleft were observed as resulting from ligand-induced conformational rearrangements of binding site residues. The first pocket (pocket A, Fig. 3A) shapes the sixth coordination site of the iron-heme and is mostly defined by residues of the small domain such as Tyr126, Cys129, Val130, Phe163, Phe164, Gly262, and Ala264. The second pocket (pocket B, Fig. 3A) is located at the entry of the catalytic site and is composed of residues such as Phe226, Phe227, Arg231, Ile354 and Leu384. Co-crystallized inhibitors specifically occupy pocket A or both pockets of the catalytic cleft (Fig. 3B). Overall, the above observations suggest that multiple ligand-induced conformations of the catalytic site may exist, favoring the molecular recognition of different structural classes of inhibitors on the part of the enzyme.

## Structure–activity relationships of IDO1 inhibitors

At odds with the paucity of compounds entered in clinical settings as drug candidates targeting IDO1, a large number of small molecules have been reported as inhibitors of the catalytic function of the enzyme. These compounds have been comprehensively reviewed in the literature with a number of survey articles.^2^,^19^–^23^ From a mechanistic point of view, IDO1 inhibitors have been reported with a competitive, non-competitive, uncompetitive or mixed mechanism of inhibition, albeit some of them lack data on the mechanism of inhibition. These definitions are based on the inhibition kinetics shown by these compounds in biochemical assay. Specifically, competitive inhibitors are defined as substrate analogues that bind to the catalytic cleft of the active ferrous form of IDO1, competing with the molecular recognition of l-Trp (**1**). Although non-competitive inhibitors are generally thought to interact with allosteric sites of the unbound and substrate-bound enzyme, non-competitive inhibitors of IDO1 were experimentally observed to bind to the catalytic cleft of the inactive ferric form of the enzyme, engaging the ferric form of the heme group in a coordinative interaction.^25^,^27^ Uncompetitive inhibitors are compounds that bind with maximal affinity to the substrate-bound complex of IDO1. However, the interpretation of kinetic studies of inhibitors suffer from the complexity of the catalytic mechanism of the enzyme which follows a redox activation cycle, and a steady-state kinetic model being composed of two substrates (l-Trp and oxygen) and two substrate recognition routes which lead to ternary complex formation.^39^ Specifically, the redox activation cycle of the enzyme consists of a shift from a ferric inactive form to a ferrous catalytically active form of the heme group. This may affect inhibition kinetic studies of uncompetitive compounds showing similar binding affinity towards both ferric and ferrous forms of IDO1, and non-competitive compounds displaying preferential binding affinity towards the inactive ferric form of the enzyme.^40^,^41^ With regard to substrate recognition routes, in the main faster route, oxygen binds first to the active ferrous form of IDO1 followed by l-Trp (**1**). In the second slower route, the order of substrate recognition is inverted with l-Trp (**1**) binding first to the enzyme followed by the interaction of oxygen. Although both routes contribute to the overall rate of catalysis, increasing the concentration of l-Trp (**1**) favors the contribution of the second route and eventually leads to substrate inhibition.^39^

In this framework, IDO1 inhibitors may show a competitive inhibition mode with respect to oxygen, being not-competitive with respect to l-Trp (**1**). Both the redox activation cycle and the steady-state kinetic model of IDO1 can be in turn affected by differences in biochemical assay conditions, eventually influencing diverse outcomes of inhibition kinetic modes.^23^

In this section, we focus the discussion on selected classes of inhibitors for which structure–activity relationships are available and have paved the way to advancements in the medicinal chemistry of IDO1, contributing to the generation of drug candidates in clinical trials (**3–7**, Fig. 1).

### Competitive inhibitors

**Fig. 4:**
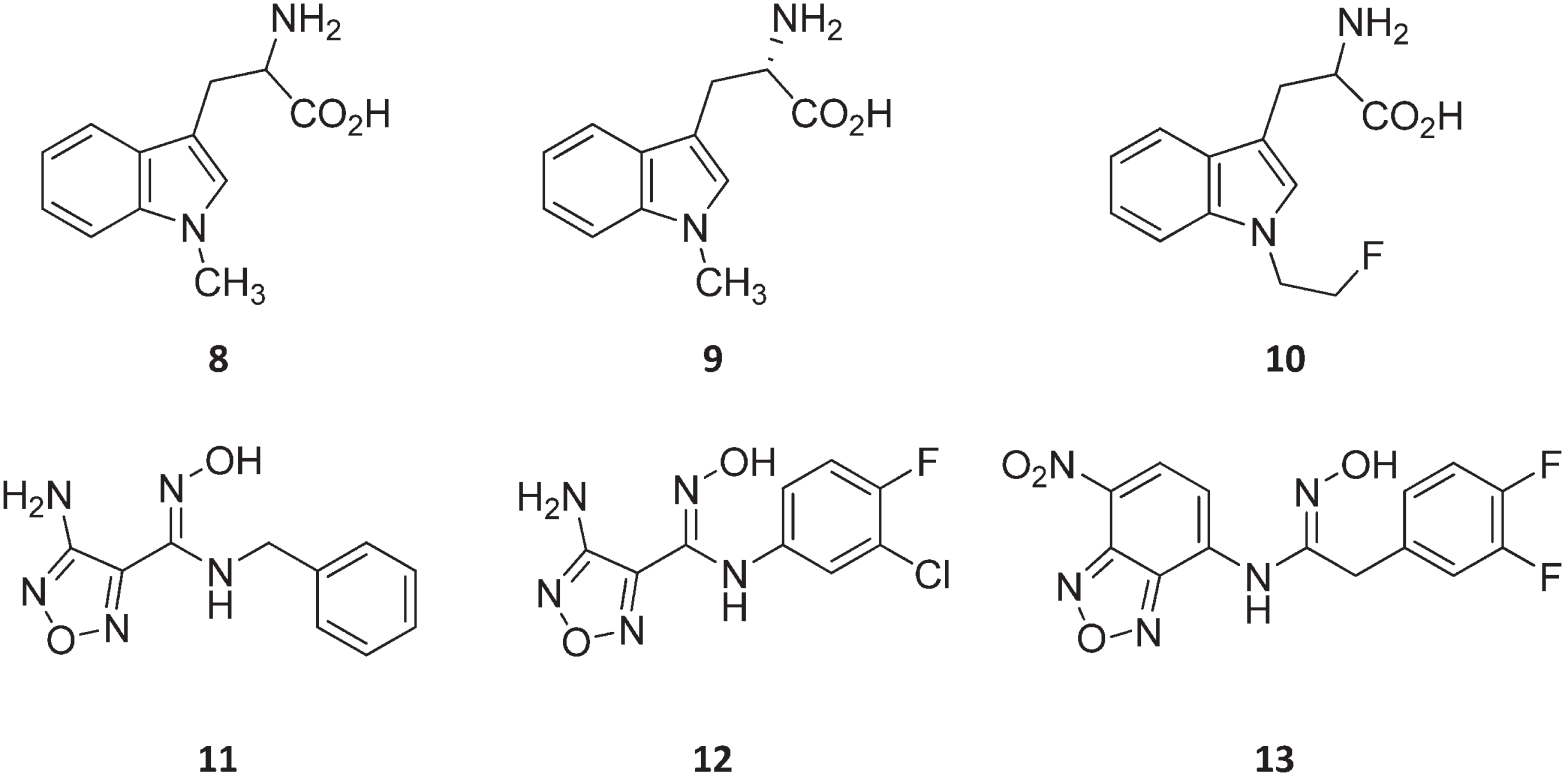
Chemical structures of slow substrates (**8–10**) and competitive inhibitors (**11–13**).

Design of l-Trp analogues was the early ligand-based strategy pursued to develop competitive inhibitors of IDO1.^42^–^44^ In this framework, proton abstraction of the indole NH group was envisaged as the pivotal event for the oxidative cleavage of l-Trp (**1**) by IDO1. The outgrowth was the *N*-methyl alkylation of the indole ring that yielded 1-methyl-Trp (1MT, **8**, Fig. 4) as a micromolar competitive inhibitor of the enzyme.^42^ While the racemic mixture of 1-MT (**7**) was reported to inhibit IDO1 with a *K*_i_ of 30 μM, the *S*-enantiomer (l-1-MT, **9**, Fig. 4; *K*_i_ = 18.0 μM; hIDO1 63% inhibition at 100 μM) was found to be more active than the *R*-stereoisomer (d-1-MT, **3**; hIDO 12% inhibition at 100 μM) in biochemical and cellular assays.^4^,^43^–^45^ Notwithstanding, some authors observed superior *in vitro* T-cell activation efficacy and *in vivo* anticancer activity for d-1-MT (**3**).^46^ The compound was further found to exert tryptophan mimetic functions, suppressing downstream IDO1 mediated effects on the mechanistic target of the rapamycin (mTOR) signalling pathway and amino acid-sensing pathway.^47^ Founded on these observations, d-1-MT (**3**, indoximod) was advanced in phase I clinical trials in combined therapy with standard chemotherapeutic anticancer drugs. Some results have been recently published, suggesting favorable pharmacokinetic and toxicity profiles.^48^ Notably, no effect of the compound was observed on plasma kynurenine levels, supporting the hypothesis that the mechanism of action of d-1-MT (**3**) may occur downstream of the IDO1 signaling pathway or may not involve IDO1 modulation. Authors, however, did not rule out that changes in kynurenine levels could be more prominent in areas not amenable to serial sampling such as tumor draining lymph nodes during d-1-MT treatment. More recently, a patent application has been filed with salts and prodrugs of d-1-MT (**3**) that have been designed to enhance systemic exposure and the plasma concentration of the inhibitor.^49^

**Fig. 5:**
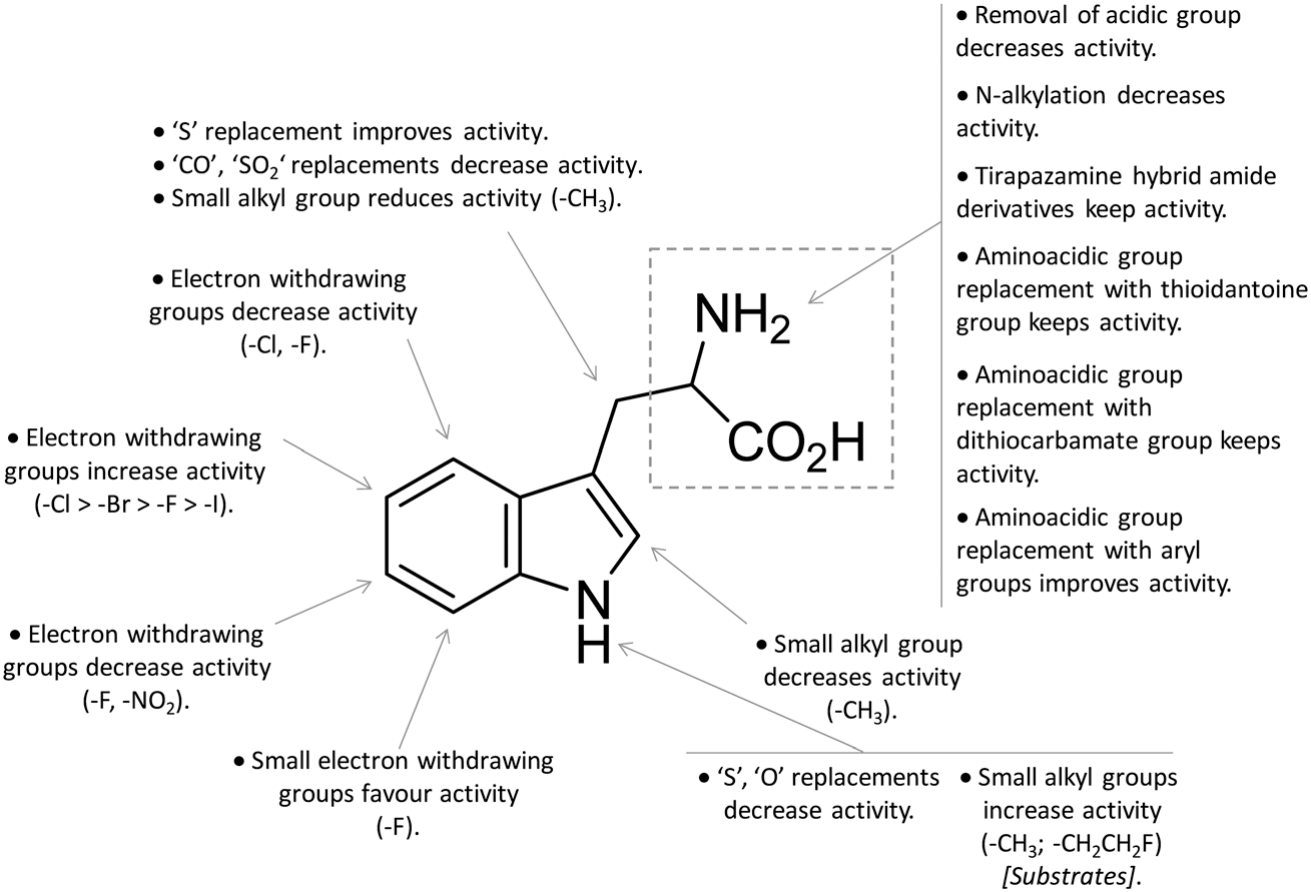
Structure–activity relationships of indole-based inhibitors originating from ref. 42, 44, 57 and 59–63.

In contrast to compound **3**, l-1-MT (**9**) has not reached clinical settings, but is still used in preclinical studies as a reference tool compound to investigate IDO1 biology in the immune system and cancer disease.^45^,^50^,^51^ Recent works suggested that l-1-MT (**9**) is a slow substrate rather than a competitive inhibitor of the enzyme.^52^,^53^ These findings have contributed to advance the understanding of the catalytic mechanism of IDO1 that is not consistent with the early proposals of a base-catalyzed abstraction mechanism, but is more in agreement with the formation of a ferryl intermediate during the catalytic turnover.^54^–^56^ Furthermore, they have been instrumental in influencing the design of 1-*N*-[^11^C]methyl-l-tryptophan ([^11^C]-**9**) and the 1-*N*-fluoroalkyl tryptophan derivative (**10**, Fig. 4) as selective substrates of IDO1 over TDO2, thereby paving the way to the development of radioactive isotopomers for the imaging study of IDO1 activity in tumoral and inflammatory tissues.^57^,^58^ A number of attempts have been made to improve the inhibitory potency of 1-MT (**7**) by screening libraries of indole-containing compounds, working with replacements of the aminoacidic side chain and/or insertion of substituents on the indole ring.^59^–^63^ Although none of these previous studies proved successful in disclosing IDO1 competitive inhibitors with submicromolar activity, they have been instrumental in drawing a structure–activity relationship scheme around the tryptophan scaffold, as depicted in Fig. 5. In particular, insertion of electron withdrawing groups on the indole ring improves the inhibition activity of parent compounds only when occurring at the C6 or C8 position, with the C6 position tolerating only a small substituent such as a fluorine atom.^44^,^63^ Replacement of the indole ring with benzothiophene or benzofurane is detrimental for activity,^42^ whereas alkylation is allowed for activity at the indolic nitrogen atom (**3**, **8–10**)^42^,^57^ but not at the C1 position.^63^ Germane to the side chain of tryptophan, few chemical manipulations account for the improvement of the inhibition activity, including the replacement of the Cβ atom with a sulfur atom and the substitution of the α-aminoacidic group with aryl moieties.^63^ As a general consideration, it cannot be ruled out that, like l-1-MT, some of these compounds may rather act as slow substrates and not be true inhibitors of IDO1.

**Fig. 6:**
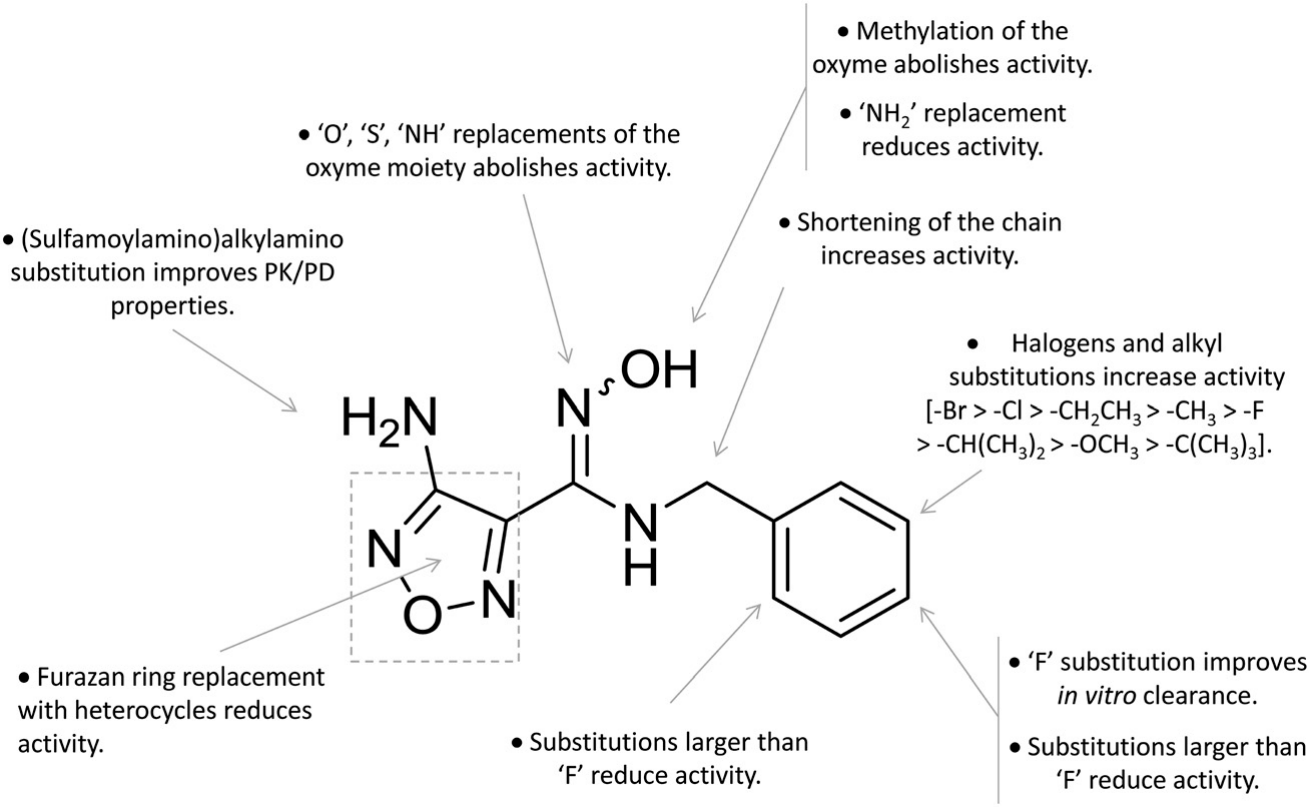
Structure–activity relationships of hydroxyamidine-based inhibitors originating from ref. 64 and 65.

A high throughput screening campaign led researchers at Incyte Corporation to identify a hydroxyamidine derivative (**11**, Fig. 4) as a hit compound in HeLa cellular assay (HeLa–IDO1 IC_50_ = 1.0 μM), on the way to developing potent and selective non-indolic IDO1 inhibitors with suitable physicochemical properties for *in vivo* studies.^64^ While absorption spectroscopy suggested direct binding of this compound to the active ferrous form of the enzyme, first efforts of hit to lead optimization were engaged to generate a focused library of hydroxyamidine analogues. The structure–activity relationships of this class of competitive inhibitors are summarized in Fig. 6. In particular, improvements of the inhibition activity from first round analogues are observed with shortening of the benzyl chain and insertion of bulky halogen or alkyl groups at the *meta* position of the phenyl ring.

**Fig. 7:**
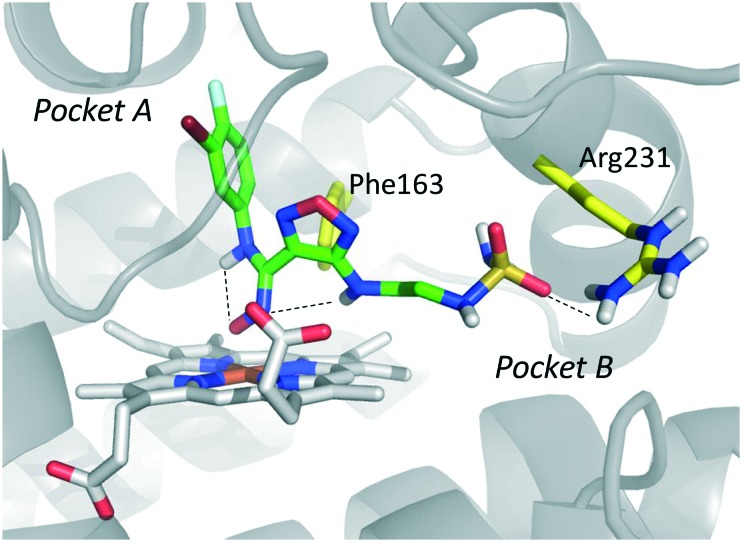
Proposed binding mode of epacadostat (**4**) to IDO1 as a result of the docking study reported in ref. 65. Intra-molecular hydrogen bonds of **4** are shown with dashed black lines.

Among library compounds, analogue **12** (Fig. 4) was found as a nanomolar inhibitor of IDO1 in HeLa cellular assay (HeLa–IDO1 IC_50_ = 0.019 μM). Such a remarkable inhibitory potency of the hydroxyamidine compound was ascribed to a dative bond of the hydroxyl group to the ferrous iron heme, perhaps mimicking the ferryl intermediate in the IDO1 catalytic turnover (Fig. 7). *In vivo* studies of lead compound **12** provided the proof of concept that the hydroxyamidine derivative was able to reduce kynurenine levels in plasma and inhibit tumor growth. Due to a poor pharmacokinetic profile for oral administration, compound **12** was further investigated through ADME studies, including metabolic stability and protein binding free fraction. As a result, phase-II glucuronidation reaction at the oxygen atom of the hydroxyamidine moiety was found as the major limiting factor for oral bioavailability of **12**. Accordingly, second rounds of compound optimization were undertaken for lead compound **12**.^65^ Since structure–activity relationships of first round optimization evidenced better inhibition potency for a bulky halogen at the *meta* position of the phenyl ring (Fig. 6), a bromine atom was preferred over a chlorine substituent at this position in second round optimization studies. Replacement of the furazan ring with heterocycles was also attempted, but resulted in being detrimental for the activity. The C3 position of the furazan moiety was then targeted with the insertion of tertiary and secondary amine substituents, envisaging the possibility to reduce the propensity to glucuronidation reaction by providing steric and/or electronic hindrance from this position to the proximal glucuronidase active site. Although the tertiary amino–furazan derivative proved inactive, secondary amine hydrophobic substituents such as methyl, ethyl and butyl groups were tolerated for biochemical inhibition activity, but did not improve the metabolic stability of the parent compound and reduced the inhibition potency in HeLa cellular assay due to a poor protein binding free fraction. Based also on docking studies into IDO1 that evidenced the occupancy of the solvent exposed pocket B of the catalytic cleft for the secondary amine ethyl group (Fig. 7), different hydrophilic capping moieties were inserted such as sulfonamide and sulfamide groups, leading to improved cellular potency and, more importantly, increased metabolic stability.

Collectively, these efforts enabled researchers to develop epacadostat (**4**, INCB024360, Fig. 1), an orally active and selective hydroxyamidine inhibitor of IDO1.^65^ It is worth noting that crystallographic studies of **4** evidenced two intramolecular hydrogen bonds between the aniline group and the oxygen atom of the hydroxyamidine moiety, and between the imino nitrogen of the hydroxyamidine and the secondary amine group of the furazan ring (Fig. 7). Authors proposed that this extensive network of intramolecular hydrogen bonds could likely account for the good cellular permeability and pharmacokinetic profile of epacadostat (**4**). Compound **4** proved to be able to reduce plasma kynurenine levels in C57BL/6 mice and to inhibit the tumor growth in mice bearing CT26 colon carcinomas, showing a 56% tumor growth control at a dose of 30 mg kg^–1^. No safety alerts were found in toxicological parameters including signs of autoimmunity. These data supported the advancement of epacadostat in clinical trials for advanced cancers, and some data have been recently published.^66^–^68^ Results highlight that epacadostat (**4**) is a well-tolerated compound and reduces plasma kynurenine levels in patients with a maximal inhibition of IDO1 at doses higher than 100 mg twice daily. The compound is actually being investigated in phase III clinical trials, in combination therapy with cancer vaccines or immune checkpoint inhibitors such as nivolumab, durvalumab, atezolizumab and pembrolizumab.

A series of nitrobenzofurazan compounds have also been reported in the literature as a result of optimization efforts of the hydroxyamidine lead compound **12** (Fig. 4).^69^ In agreement with the structure–activity relationships of hydroxyamidine derivatives (Fig. 6), the authors found that incorporation of a methylene group between the aryl ring and the hydroxyamidine moiety resulted in a slight decrease of the inhibitor activity against IDO1 in biochemical assay, whereas insertion of halogens in the aryl ring favored the inhibition potency. Among the tested compounds in the MDA-MB-231 breast cancer cell line, the authors found nitrobenzofurazan derivative **13** as the most potent competitive inhibitor of the series, with a cellular IC_50_ of 50 nM and selectivity over TDO2 inhibition. No *in vivo* efficacy studies are, however, reported for nitrobenzofurazan compounds.

More recently, a novel IDO1 inhibitor (PF-06840003, **5**, Fig. 1), developed by researchers at iTeos Therapeutics and licensed to Pfizer, has entered phase I clinical trials for the treatment of patients with malignant gliomas. *In vivo* preclinical studies have shown that PF-06840003 (**5**) is able to reduce intratumoral kynurenine levels by >80% and arrest tumor growth in multiple syngeneic models, in combination with immune checkpoint inhibitors.^70^ Unfortunately, very few data are available in the literature about discovery and structure–activity relationships of this compound. However, on the basis of its indole-based structure and recent patents filed by iTeos, compound **5** may likely be a competitive inhibitor of IDO1.^71^

### Non-competitive and uncompetitive inhibitors

**Fig. 8:**
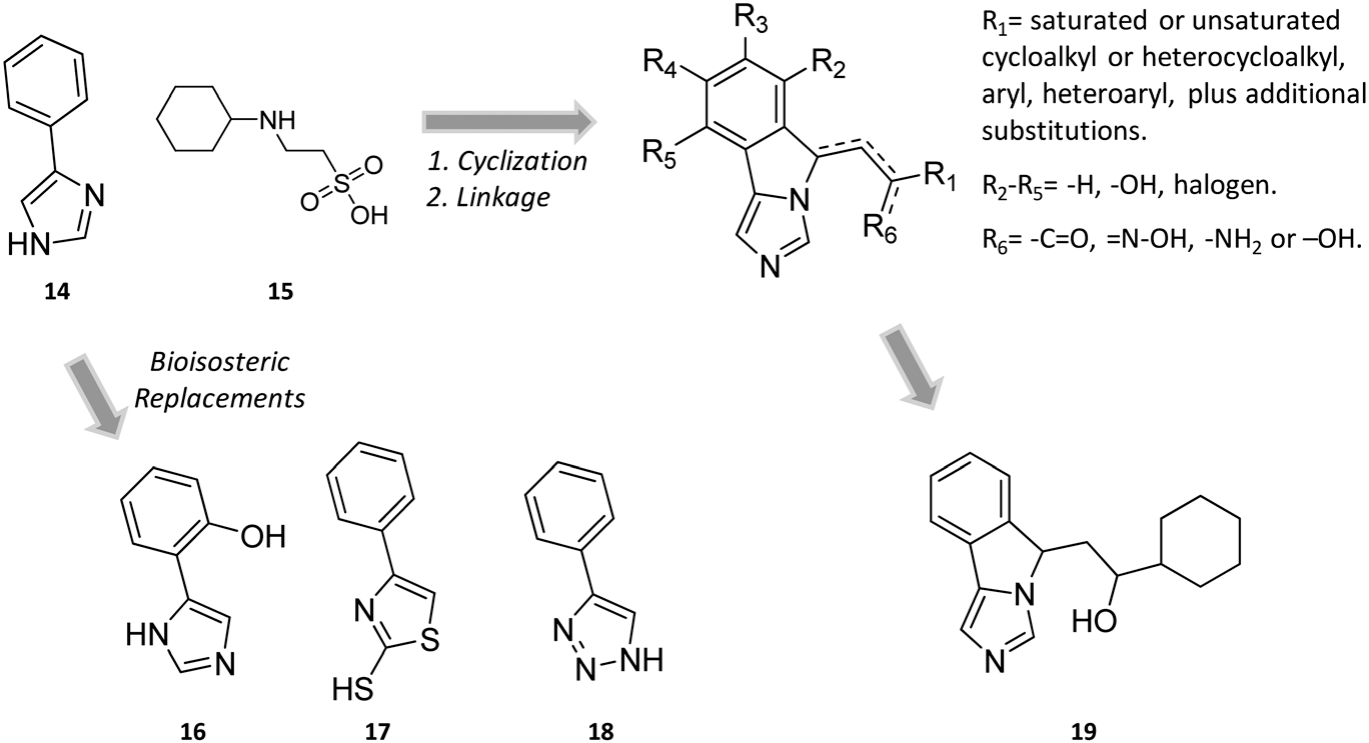
Chemical structures of non-competitive/uncompetitive inhibitors (**14**, **16–19**) and relative structure-based design strategies. The Markush structure of NLG919 analogues is taken from ref. 78.

**Fig. 9:**
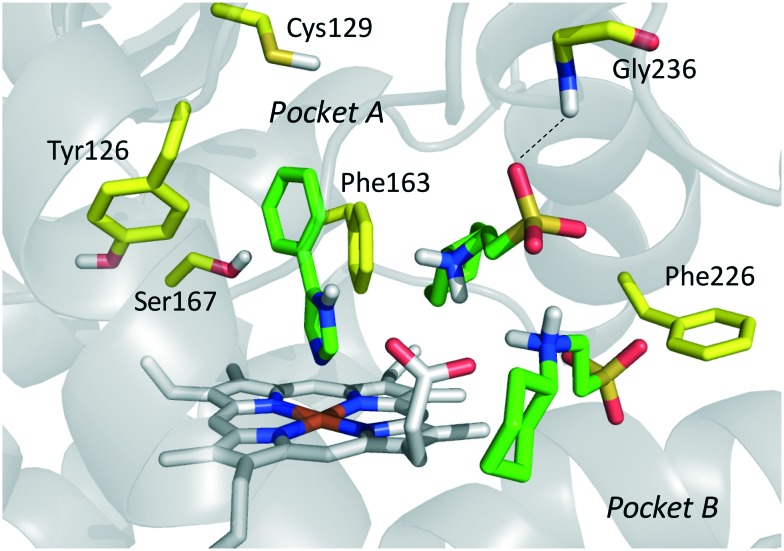
Binding mode of 4-PI (**14**, shown in green carbon atom sticks) to IDO1 resulting from crystallographic studies (pdb code: ; 2D0T). Key residues for structure–activity relationships are labeled and shown in yellow carbon atom sticks. CHES (**15**) molecules binding to the catalytic cleft are also shown in green carbon atom sticks.

In the seminal paper of Sono and Cady, 4-phenylimidazole (4-PI, **14**, Fig. 8) was discovered as a weak non-competitive inhibitor of IDO1, showing a preferential binding to the inactive ferric form of the enzyme.^40^ Almost two decades later, this compound was used to solve the first ligand-bound crystal structure of IDO1 which demonstrated a direct interaction of 4-PI (**14**) with the sixth coordination site of ferric heme, and the engagement of aromatic residues through π-stacking interactions (Tyr126, Phe163, Fig. 9).^25^ The inspection of the ligand-bound complex thus confirmed early suggestions that the non-competitive inhibition mechanism of 4-PI (**14**) has to be ascribed to the impairment of the reductive activation of IDO1 rather than to interactions with an accessory site of the protein. Analysis of the crystal structure further unveiled the presence of additional molecules from the crystallization buffer, namely 2-(*N*-cyclohexylamino)ethane sulfonic acid (CHES, **15**, Fig. 8), which were bound to pocket B of the catalytic cleft (Fig. 9). Later studies suggested that the pocket holding these molecules may define an accessory site for substrate inhibition, effector or uncompetitive inhibitor binding to IDO1.^33^,^34^,^53^,^72^

**Fig. 10:**
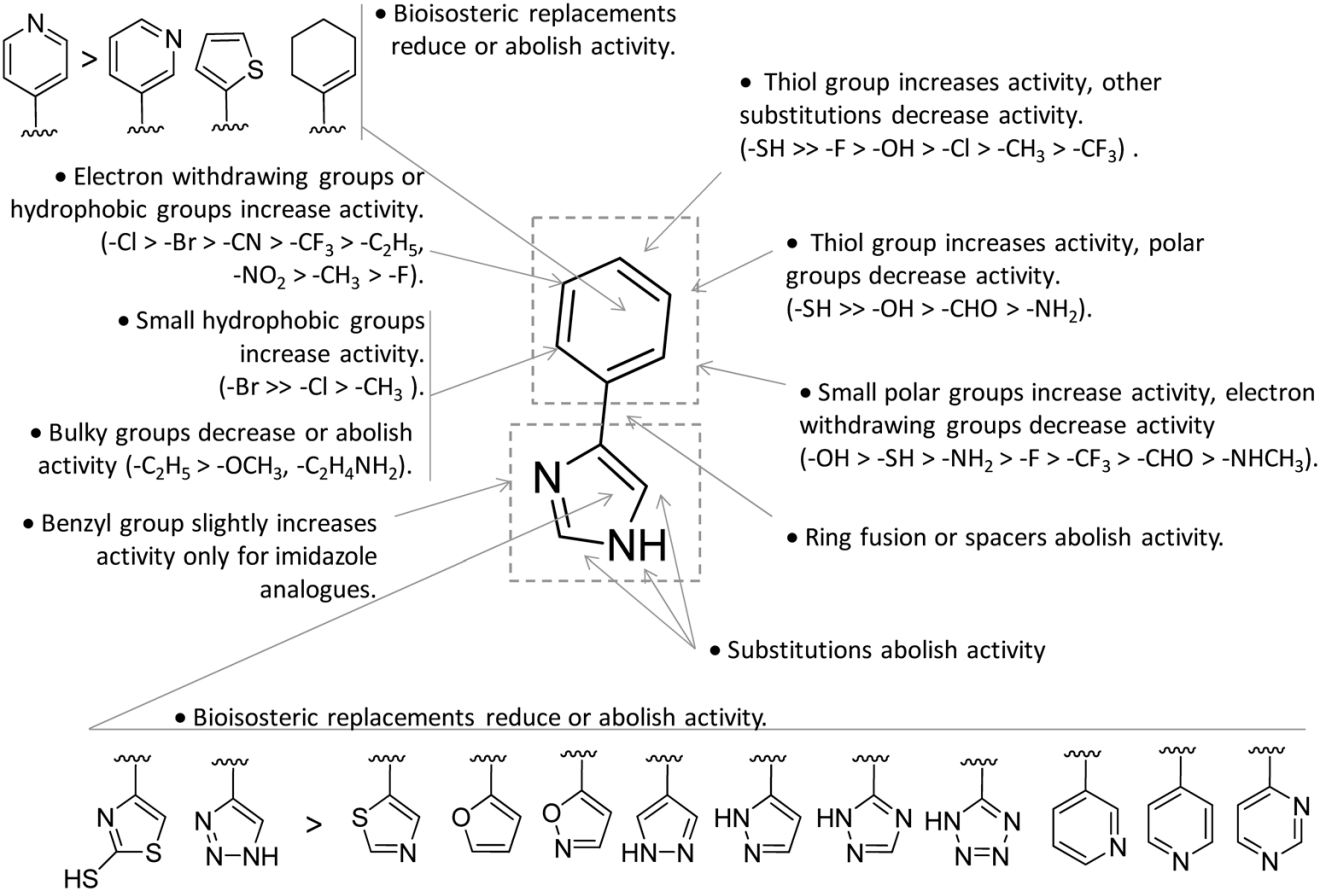
Structure–activity relationships of 4-PI-based inhibitors originating from ref. 73–76.

Collectively, these results constituted invaluable early insights into the IDO1 structure which have boosted structure-based drug design strategies for novel enzyme inhibitors. Among the first applications, researchers engaged in hit to lead optimization efforts of 4-PI (**14**).^73^ Exploiting the binding mode of 4-PI (**14**) to IDO1, they followed three routes of analogue design. With the aim of gaining interactions with Phe163, Phe226 and/or the propionate group of the heme cofactor, the first route consisted in inserting mostly an amino-alkyl or benzyl group on the N3 or N1 position, and on the C2 position of the imidazole ring. As a result, only the N3 benzyl analogue proved slightly more active (IC_50_ = 32 μM) than the parent compound (4-PI, **14**, IC_50_ = 48 μM), suggesting the successful engagement of aromatic residues in π-stacking interactions. The second structure-based optimization route was directed at engaging Cys129 and Ser167 in hydrogen bonds with specific polar group functionalization of the phenyl ring of 4-PI (**14**). It was found that hydroxyl groups at *ortho* positions (IC_50_ = 5.3 μM), or a thiol group at the *meta* (IC_50_ = 7.6 μM) or *para* (IC_50_ = 7.7 μM) position led to improved inhibitory potency. Of note, quantum mechanical calculations excluded inductive electronic effects of such substituents on the charge of the N1 atom, supporting the idea that the improvement of activity was ascribed to specific hydrogen bond interactions with Cys129 and/or Ser167. In this regard, it is worth noting that a very recent mutagenesis study has confirmed the importance of Ser167 for the potency of 4-(*ortho*-hydroxyl)-PI (**16**, IDO1^WT^ IC_50_ = 1.2 μM, IDO1^S167A^ IC_50_ = 41 μM; Fig. 8), supporting the idea of a hydrogen bond between such a residue and the hydroxyl group at the *ortho* position.^36^ Germane to the third optimization route, replacement of the imidazole group was attempted to probe the interaction with the sixth coordination site of the heme iron. Although 5-substituted-thiazole, 3- or 4-substituted-pyrazole, 2-substituted-furan and 3-substituted-pyrimidine moieties were used as bioisosteric substituents, no improvement of the inhibitory activity was observed.^73^ In a successive structure-based screening campaign, other authors found hit compounds that structurally resemble 4-PI, thereby extending the structure–activity relationships around this chemical scaffold (Fig. 10).^74^ Specifically, 2-thiol-4-phenyl-thiazole (**17**, Fig. 8) and 4-phenyl-triazole (**18**) were disclosed as moderately active IDO1 inhibitors in biochemical assays. However, in contrast to the triazole derivative (**18**, IC_50_ = 60 μM; HEK293 IC_50_ = 70 μM), 2-thiol-4-phenyl-thiazole exhibited low micromolar inhibitory activity in cellular assay (**17**, IC_50_ = 50 μM; HEK293 IC_50_ = 4.0 μM). The imidazole group replacement of 4-PI (**14**) with the triazole ring was also exploited in subsequent studies to generate focused libraries of 4-aryl-1,2,3-triazoles as IDO1 inhibitors.^75^,^76^ While results confirmed that substitutions at the *para* position of the phenyl ring were generally detrimental for IDO1 inhibitor activity, they further suggested some substituents that are allowed at the *meta* position of the phenyl ring such as chlorine (IC_50_ = 1.2 μM; P815B IC_50_ = 0.62 μM) and bromine (IC_50_ = 2.0 μM; P815B IC_50_ = 0.94 μM). Germane to the *ortho* position, it was again suggested that a small polar moiety such as the hydroxyl group improves the inhibitor potency (IC_50_ = 15 μM; P815B IC_50_ = 1.7 μM), while large bulky groups are detrimental for the activity.

Notwithstanding, some differences were also observed with respect to early structure–activity relationships of 4-PI analogues (Fig. 10).^73^ At odds with imidazole-based analogues, it was found that insertion of a benzyl group at the N3 position of the triazole ring was detrimental for the activity.^76^ Furthermore, replacement of the phenyl ring with a *para* pyridine moiety was less detrimental for the inhibitor activity of the triazole-based derivative (IC_50_ = 85 μM) than the imidazole-based analogue (IC_50_ = 1800 μM). Collectively, the similarities and discrepancies between the two structurally related classes of inhibitors were explained by the presence of a conserved ligand binding mode in the catalytic cleft, but distinct electronic features affecting the interaction with the heme cofactor. In this regard, quantum mechanical calculations, p*K*_a_ determinations and Hansch analysis suggested that triazole-based analogues interacted with IDO1 in the deprotonated form, and electron-withdrawing groups on the phenyl ring influenced the coordination bond to the heme iron. As weaker acidic compounds, imidazole-based derivatives bound to the enzyme in the neutral form, and electron-withdrawing groups of the phenyl ring did not have strong effects on the coordinative interaction with the heme iron.^76^ A switch from a non-competitive mechanism to an uncompetitive mechanism of inhibition was noticed in biochemical assays for some imidazole-based compounds and triazole-based derivatives with respect to the parent compound 4-PI (**14**).^73^,^75^ This observation was tentatively explained with these compounds showing a similar binding affinity towards both the inactive ferric form and the active ferrous form of IDO1.^73^

Still more was to come after researchers at Newlink Genetics combined cyclization and linkage approaches on 4-PI (Fig. 8), leveraging the presence of CHES (**13**) in the ligand-bound crystal structure of IDO1 (Fig. 9). In particular, they devised a library of fused imidazole derivatives as IDO1 inhibitors.^77^ Although scant data are available on this class of compounds in the literature, NLG919 (GDC0919, **6**, structure undisclosed) was first reported at the 104th Annual Meeting of the American Association for Cancer Research as the most interesting compound of the series.^78^ The compound proved a potent orally bioavailable IDO1 inhibitor (*K*_i_ = 7 nM; EC_50_ = 75 nM), showing favorable pharmacokinetic and toxicity profiles. When orally administered in mice, NLG919 reduced plasma kynurenine levels and was able to enhance the anti-tumor response of resting pmel-1 T cells to vaccination with cognate hgp100 peptide in mice bearing B16F10 tumors. The compound is now being evaluated in clinical trials for safety and preliminary efficacy in patients with advanced solid tumors, as stand-alone therapeutic intervention or in combination therapy with atezolizumab. Another compound (NLG919 analogue **19**, Fig. 8) of the fused imidazole library was used as a chemical tool by other academic groups to validate high-throughput screening assay for IDO1 inhibition,^79^ develop immunostimulatory nanomicellar carriers,^80^ solve additional ligand-bound crystal complexes of the enzyme and investigate structure–activity relationships.^27^ It should be mentioned that the chemical structure of **19** could be identical to GDC0919 (**6**), as reported in some catalogues of commercially available inhibitors.^81^

**Fig. 11:**
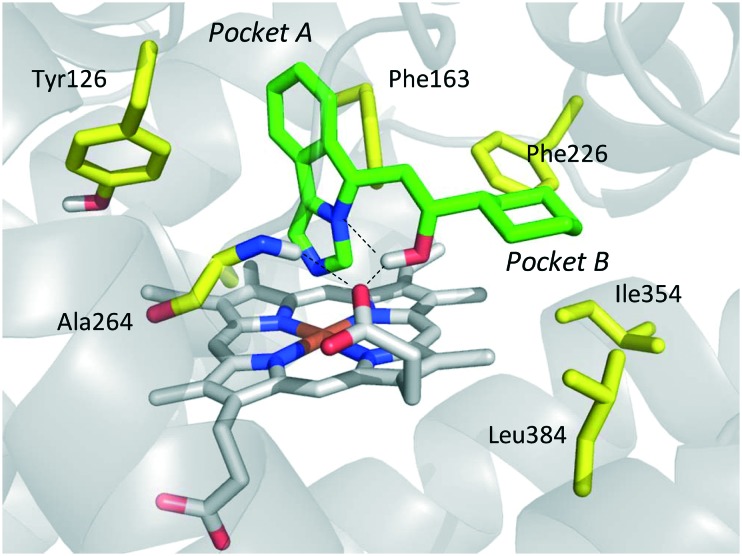
Binding mode of the NLG919 analogue (**19**, shown in green carbon atom sticks) to IDO1 resulting from crystallographic studies (pdb code: ; 5EK3). Key residues are labeled and shown in yellow carbon atom sticks. Inter- and intra-molecular hydrogen bonds of **19** are shown with dashed black lines.

In the crystallization study, the inhibitor activity of the analogue **19** was determined in the nanomolar range of potency (IC_50_ = 38 nM).^27^ In agreement with the binding mode of 4-PI (**14**, Fig. 9), inspection of the ligand-bound complex revealed a direct interaction of the distal nitrogen atom of the fused imidazole with the sixth coordination site of ferric heme (Fig. 11).

**Fig. 12:**
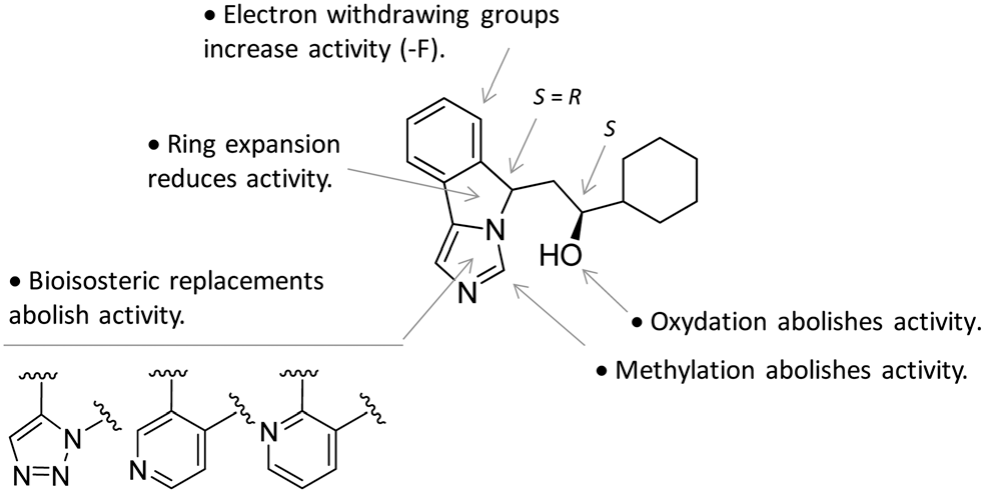
Structure–activity relationships of NLG919-based inhibitors originating from ref. 27.

In particular, the imidazoleisoindole group adopts a binding mode similar to 4-PI (**14**), occupying pocket A of the catalytic cleft over the heme plane, whereas the cyclohexylethanol moiety extends into pocket B engaging residues Phe226, Ile354, and Leu384 in hydrophobic interactions. Key intermolecular and intramolecular hydrogen bonds were also observed involving the isoindole nitrogen, the cyclohexylethanol group, one heme propionic moiety and the backbone atoms of Ala264. Remarkably, being a mixture of four stereoisomers, the analysis of the electron density map suggested that both (*R*, *S*) and (*S*, *S*) stereoisomers could nicely fit the map, providing similar interactions with IDO1 (Fig. 11 and 12).

This observation was supported by chiral chromatography separations and biochemical assays, showing similar IC_50_ values of 19.5 nM and 28.9 nM for the two stereoisomers. Furthermore, the design of a small library of NLG919 analogues yielded structure–activity relationships in part resembling those of 4-PI analogues (Fig. 12).

**Fig. 13:**
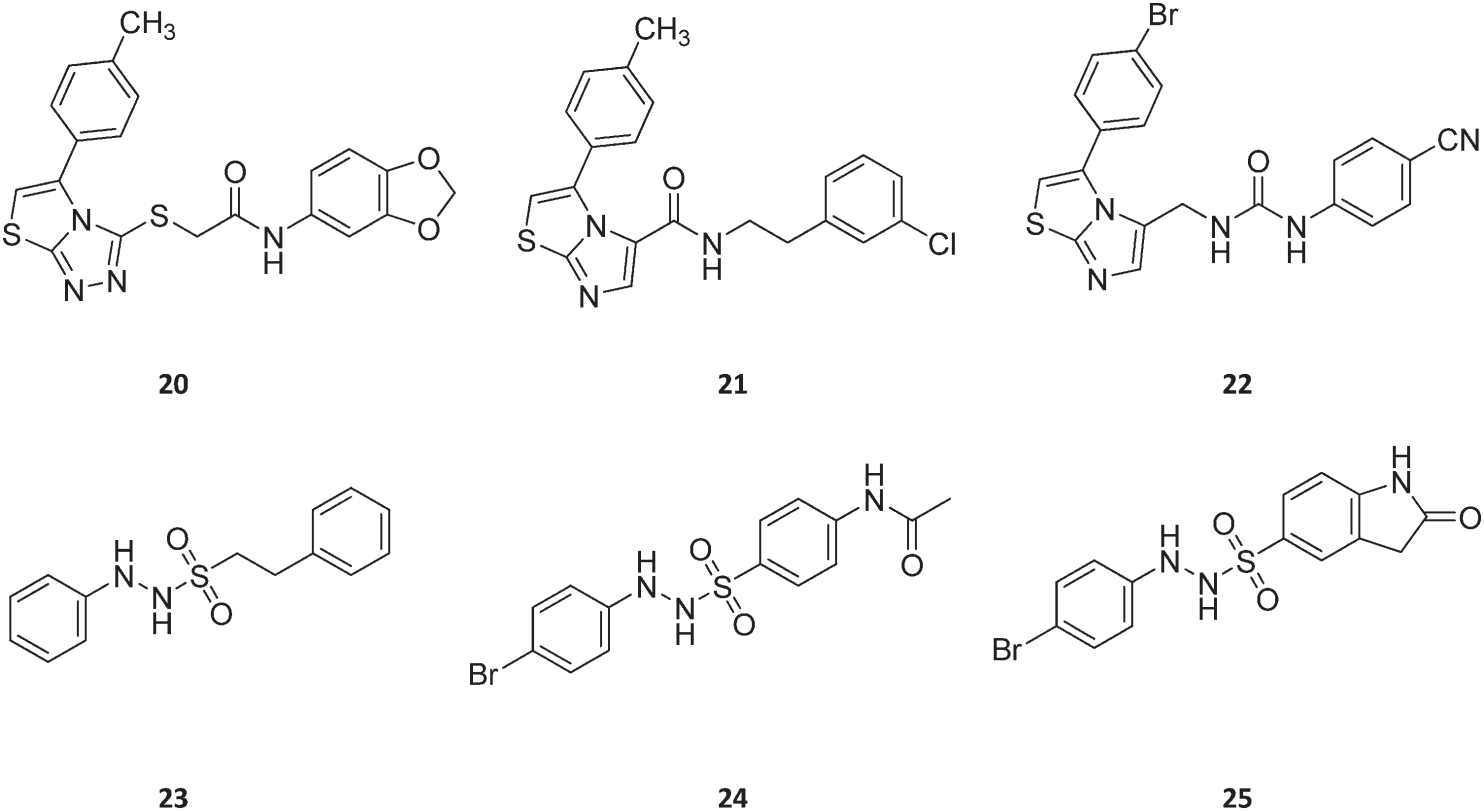
Chemical structures of IDO1 inhibitors (**20–25**) with a hypothetical non-competitive and/or uncompetitive mechanism of inhibition.

Screening of a proprietary collection of compounds led researchers at Amgen to discover compound Amg-1 (**20**, Fig. 13) as a selective IDO1 inhibitor over TDO2 and IDO2, with an IC_50_ of 3.0 μM as determined using the Bridge-IT tryptophan fluorescence assay.^82^

**Fig. 14:**
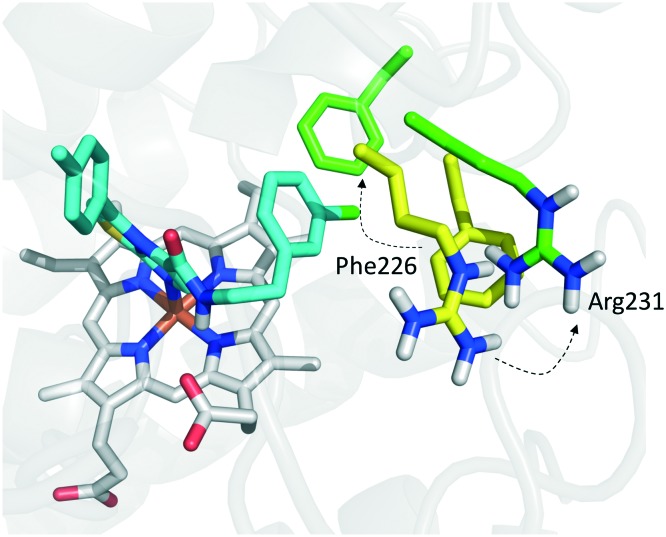
Binding mode of imidazothiazole derivative **21** (shown in green carbon atom sticks) to IDO1 resulting from crystallographic studies (pdb code: ; 4PK6). Phe226 and Arg231 are labeled and shown in sticks. Ligand-induced conformational changes are highlighted with dashed arrows from positions observed in the Amg-1 (**20**) bound crystal structure of IDO1 (Phe226 and Arg231 shown in yellow atom sticks, pdb code: ; 4PK6) to actual positions in the ligand **21** bound complex (Phe226 and Arg231 shown in green atom sticks, pdb code: ; 4PK5).

Founded on crystallographic studies of Amg-1 (**20**) in complex with IDO1, other researchers engaged in the design and synthesis of imidazothiazole derivatives as IDO1 inhibitors.^26^ These studies were pioneering in depicting the first structure–activity relationship scheme for moieties occupying pocket B of the enzyme, and further extending the structure–activity relationships of substituted phenyl groups binding to pocket A of the catalytic cleft (Fig. 10). Specifically, it was found that linking the imidazothiazole core nucleus to the substituted aromatic side chain with an amide group yielded inhibition activities in the same micromolar range as of Amg-1 (**21**, IC_50_ = 1.9 μM). Conversely, the introduction of a linearly rigid linker, such as the urea group, increased the activity to the nanomolar range of potency (**22**, IC_50_ = 0.077 μM). These observations were explained with further crystallization studies on the amide linked imidazothiazole derivative **21** that unveiled a ligand-induced conformational change of Phe226 along with a torsional bend of the ligand side chain, eventually resulting in the loss of a key interaction with Arg231 and the engagement of Phe226 in hydrophobic interactions (Fig. 14).

Germane to the phenyl ring binding into pocket A, the preference of bulky halogens, such as bromine, for enzyme inhibition when inserted at the *para* position was observed. Although no inhibition kinetic study is reported in the literature for these compounds, the observed direct interaction of the imidazothiazole moiety with the heme iron would suggest a non-competitive and/or uncompetitive mechanism of inhibition.

The results of another screening study led researchers to identify 2-phenyl benzene-ethanesulfonylhydrazide (**23**, Fig. 13) as a hit compound with potent IDO1 inhibition activity in biochemical assay (IC_50_ = 167 nM), but poor cellular activity (HeLa EC_50_ > 10 μM).^83^ The first round of hit to lead optimization studies yielded lead compound **24**, bearing an acetamido moiety at the *para* position of the benzenesulfonyl group, and a bromine atom at the *para* position of the phenyl hydrazine group. This compound proved to have potent IDO1 inhibition activity in both biochemical and cellular assays (IC_50_ = 120 nM; HeLa EC_50_ = 85 nM). Further lead optimization studies were carried out around this compound to improve its pharmacodynamic and pharmacokinetic profiles.^84^ Specifically, the optimization strategy consisted in the synthesis of cyclic analogues of the *para* acetamido moiety which yielded compound **25** as a potent IDO1 inhibitor with favorable drug exposure and good oral bioavailability for preclinical study in a CT26 mouse model of colorectal tumor. Oral administration of the compound by gavage at 200 mg kg^–1^ and 400 mg kg^–1^ resulted in tumor growth delay of 63% and 73%, respectively. Although no kinetic inhibition study was performed for this class of compounds, a previous work reported a direct interaction of the phenylhydrazine fragment with both ferric and ferrous states of IDO1.^85^ Hence, it is likely that compound **25** may be endowed with a non-competitive and/or uncompetitive mechanism of inhibition.

Finally, it should be mentioned that several other chemical classes of non-competitive, uncompetitive or mixed IDO1 inhibitors have been reported in the literature, including natural products and synthetic compounds from screening of chemical libraries. While some of these classes have proved suboptimal for clinical development, others have pharmacological profiles pending to be confirmed in *in vivo* studies, and/or very few structure–activity relationship data available in the literature.^2^,^19^–^23^

## Outlook and conclusions

Formerly thought as an effector enzyme of the immune system debarring pathogen bacteria from the essential amino acid l-Trp, IDO1 has experienced a new lease of life with seminal discoveries about its involvements in mediating maternal immune tolerance and the tumor immuno-editing process. A great deal of interest has thus been devoted to this enzyme as a promising drug target for the development of novel immunomodulatory drugs for cancer disease. In this framework, medicinal chemistry efforts over the past 20 years have yielded several classes of competitive, non-competitive and uncompetitive inhibitors. Notwithstanding, only very few of these compounds have progressed in clinical trials, thereby evidencing major challenges in obtaining compounds with convincing pharmacological profiles for clinical development. In one of such successful cases (indoximod, d-1MT, **3**), it is even still questioned whether the mode of action is IDO1 dependent or rather relies on the interaction with downstream effector proteins of the signaling pathway. The growing understanding of the complexity of IDO1 biology, including its catalytic redox machinery and signaling function, is providing grounds for better design of biological assays and selection of lead compounds. Germane to these factors, while some authors have suggested recommendations to improve successful translation of *in vitro* results of IDO1 inhibitors from biochemical and cellular assays to *in vivo* pharmacological activity,^23^ we anticipate that other sources of biological evidence will prove beneficial to predict improvements in clinical development success of IDO1 inhibitors. On the one side, deployment of biophysical methods can aid the selection of high quality lead compounds, providing validation of direct target engagement and orthogonal confirmation of functional activity. These assays may include additional crystallographic studies, NMR experiments, surface plasma resonance (SPR) and/or MicroScale thermophoresis (MST) studies. The general impact and opportunities of such biophysical methods in drug discovery have been recently reviewed elsewhere.^86^ These aspects are of utmost benefit for our protein target, where substrate inhibition, sensitivity to redox-cycling compounds and the non-catalytic signaling function of IDO1 may impact the selection of poor quality inhibitors in biochemical and/or cellular assays. On the other side, beyond l-Trp consumption, development of additional assay readouts can enable more in-depth functional characterization of lead compounds, providing thorough appraisals of their modulatory effects not only on the catalytic activity, but also on the ITIM-dependent signaling function of IDO1 and/or immunoregulatory pathways mediated by kynurenine metabolites. These readouts may include quantification of enzyme phosphorylation, levels of immunoregulatory cytokines downstream to the IDO1 signaling function,^87^ and/or modulatory activities of IDO1 inhibitors against AhR functions.^88^ Notably, it is actually unknown whether clinical advanced inhibitors may differently regulate other functionally relevant states of the enzyme with regard to post-translational modification of ITIM and binding partners such as SHP-1, SHP-2, and SOCS3. In this regard, it should be mentioned that drug candidates targeting other anticancer targets, such as PARP-1, have shown a different ability to modulate the non-catalytic function of the enzyme, independently from the potency of the compound at inhibiting the catalytic activity.^89^,^90^ Likewise, inhibitors targeting JAK kinase have been reported to, depending on the binding mode, stabilize different functionally relevant states of the enzyme that are prone to lose activation-loop phosphorylation or to increase activation-loop phosphorylation, despite blocking the catalytic activity of the enzyme.^91^ A very recent work has shown that some of the advanced clinical IDO1 inhibitors (**3**, **4**, **19**) are able to act as agonists of AhR.^88^ It is our opinion that, in the near future, harnessing these kinds of studies to early stages of IDO1 drug discovery pipelines will play a key role in enabling the development of new and more innovative immunotherapeutic drugs targeting both the catalytic activity and non-catalytic function of the enzyme.

## Conflict of interest

The authors declare no competing interests.
